# Liquid-based cytology specimens for next-generation sequencing in lung adenocarcinoma: challenges and evaluation of targeted therapy

**DOI:** 10.1186/s12885-024-12520-2

**Published:** 2024-06-20

**Authors:** Xiaoyue Xiao, ZiHan Sun, Shuo Liang, Weihua Li, HuiQin Guo, Huan Zhao, LinLin Zhao, HaiYue Ma, Yue Sun, Cong Wang, XinXiang Chang, ZhiHui Zhang

**Affiliations:** Cytopathology Section, Department of Pathology, National Cancer Center/National Clinical Research Center for Cancer/Cancer Hospital, Chinese Academy of Medical Sciences and Peking Union Medical College, Beijing, 100021 China

**Keywords:** Lung adenocarcinoma, Liquid-based cytology, NGS, Targeted therapy, Tumor cellularity

## Abstract

**Background:**

To explore challenges of liquid-based cytology (LBC) specimens for next-generation sequencing (NGS) in lung adenocarcinoma and evaluate the efficacy of targeted therapy.

**Methods:**

A retrospective analysis was conducted on the NGS test of 357 cases of advanced lung adenocarcinoma LBC specimens and compared with results of histological specimens to assess the consistency. The impact of tumor cellularity on NGS test results was evaluated. The utility of epidermal growth factor receptor-tyrosine kinase inhibitors (EGFR-TKIs) was collected. Clinical efficacy evaluation was performed and survival curve analysis was conducted using the Kaplan-Meier method.

**Results:**

There were 275 TKI-naive and 82 TKI-treated specimens, the mutation rates of cancer-related genes detected in both groups were similar (86.2% vs. 86.6%). The *EGFR* mutation rate in the TKI treated group was higher than that in the TKI-naive group (69.5% > 54.9%, *P* = 0.019). There was no significant difference in the *EGFR* mutation frequency among different tumor cellularity in the TKI-naive group. However, in the TKI treated group, the frequency of *EGFR* sensitizing mutation and T790M resistance mutation in specimens with < 20% tumor cellularity was significantly lower than that in specimens with ≥ 20% tumor cellularity. Among 22 cases with matched histological specimens, 72.7% (16/22) of LBC specimens were completely consistent with results of histological specimens. Among 92 patients with *EGFR*-mutant lung adenocarcinoma treated with EGFR-TKIs in the two cohorts, 88 cases experienced progression, and the median progression-free survival (PFS) was 12.1 months.

**Conclusions:**

Cytological specimens are important sources for gene detection of advanced lung adenocarcinoma. When using LBC specimens for molecular testing, it is recommended to fully evaluate the tumor cellularity of the specimens.

**Supplementary Information:**

The online version contains supplementary material available at 10.1186/s12885-024-12520-2.

## Introduction

Lung cancer is the leading cause of cancer-related deaths worldwide [1], with non-small cell lung cancer (NSCLC) accounting for approximately 85% of cases. 70% of NSCLC patients are in advanced stages of the disease at the time of diagnosis, with adenocarcinoma being the most common histological subtype [2, 3]. Platinum-based chemotherapy is frequently used in traditional treatments for advanced NSCLC, which has a poor prognosis and a 2-year survival rate of only 11% [4]. With the advent of precision therapy, molecular testing is becoming increasingly important in diagnosis and clinical decision-making. Targeted medicines benefit patients with certain gene mutation greatly, improving overall survival (OS), PFS, and quality of life [5]. The gold standard for molecular diagnosis of lung cancer is genetic testing of histological specimens. Obtaining histological samples from advanced NSCLC patients who have missed the option for surgical intervention can be quite difficult. As a result, cytological samples are frequently the basis for making decisions about diagnosis and treatment [3]. NGS is an efficient and cost-effective method that can identify clinically actionable mutations in various genes through high-throughput sequencing [6]. However, molecular pathology laboratories still face challenges at both the technological level and those related to the tumor’s biological characteristics [7]. NGS detection of cell block specimens has been widely reported in lung adenocarcinoma [8]. However, there is a lack of extensive clinical research data on NGS detection utilizing liquid-based cytology specimens, which differ from cell block specimens in terms of fixation and preparation techniques, and the effectiveness of targeted therapies based on these findings. In this study, we conducted a retrospective research of NGS results from 357 cases of lung adenocarcinoma using liquid-based cytology specimens. We also examined the effectiveness of targeted therapies and tumor biological characteristics to explore the reliability of gene mutation detection using liquid-based cytology specimens.

## Materials and methods

### Case selection

From January 2017 to December 2021, liquid-based cytology specimens were collected from patients with lung adenocarcinoma who visited the Pathology Department of the Cancer Hospital, Chinese Academy of Medical Sciences. Through cytopathology or histology, adenocarcinoma was identified in every case. To acquire basic clinical features and follow-up information, the electronic medical record system was used to access patient medical records which included the following information: gender, age, pathological subtype, results of NGS tests, 8th edition of the American Joint Committee on Cancer (AJCC) cancer staging system, usage and effectiveness of EGFR-TKIs, and PFS, which defines as the interval between the start of treatment and the onset of disease progression or death from any cause, were all collected in this study.

### Specimen preparation

(1) Serous effusion specimens: 185 cases included pleural effusion, peritoneal effusion, pericardial effusion, cerebrospinal effusion, etc. The specimens were centrifuged, the supernatant was discarded, and the cellular layer was transferred into liquid-based preservation solution to prepare liquid-based slides.

(2) Fine needle aspiration (FNA) specimens: 141 cases included lymph nodes and other tissues, which were aspirated by using a 23G disposable syringe (needle gauge 0.7 mm), and the aspirate was used to prepare both smears and liquid-based slides.

(3) Fibreoptic bronchoscope brush specimens: 29 cases were collected. Under the fibreoptic bronchoscope, mucosal tissue was brushed and then washed into liquid-based preservation solution to prepare liquid-based slides.

### Mutation analysis by NGS

DNA was extracted using the QIAamp® DNA mini kit (Qiagen, Germany) in accordance with the manufacturer’s directions. The Qubit 2.0 fluorometer (Thermo Fisher Scientific, Carlsbad, CA, USA) was used to calculate the DNA concentration. The Ion AmpliSeq Colon and Lung Cancer Panel on the Personal Genome Machine (PGM) platform (Thermo Fisher Scientific) was used to examine the samples’ mutation status. This panel contains 92 pairs of primers targeting 22 cancer-related genes, including *EGFR, KRAS, BRAF, PIK3CA, HER2, KIT, NTRK3, PTEN, RET, CCND1, ALK, CDKN2A, CTNNB1, MET, TP53, ROS1, RB1, PIK3R1, PIK3CG, FGFR1, FGFR2*, and *FGFR3*. With the use of multiplex PCR and 10 ng of genomic DNA, each sample was connected to a different Ion Xpress barcode. Thermo Fisher Scientific’s Ion OneTouch Template Kit and Ion OneTouch ES were used to prepare the template on Ion Sphere Particles (ISPs) through library amplification after purification and equilibration. On 316 or 318 chips, the prepared ISPs were loaded before being sequenced on the PGM. With the aid of the Torrent Suite 2.0 program, signal processing, base calling, and alignment were carried out. The Integrative Genomics Viewer was used to further identify the variants after Torrent Variant Caller had annotated them. Mutations were determined when the coverage was > 1000 and the mutant allele frequency (MAF) was ≥ 5%.

### Tumor cellularity assessment

This study’s evaluation of tumor cellularity followed the methodology employed in Li et al.‘s study [9], in which each slide was independently assessed by two pathologists and the percentage of tumor cells was calculated in increments of 5%. The final tumor cell count was calculated by averaging the two pathologists’ estimates. An additional pathologist estimated the percentage of tumor cells if the gap between the two pathologists’ estimates was greater than 10%. The average of the three pathologists’ estimates was used to determine the final tumor cellularity.

### Statistical analysis

Statistical analysis was performed using R 4.3.0 software. The comparison of inter-group rates, the relationship between tumor cell proportion and mutation frequency were analyzed using the chi-square test and Fisher’s exact probability test. The consistency of the genetic testing results between LBC and histological specimens was assessed (complete inconsistency: kappa value < 0.2, moderate consistency: kappa value = 0.21–0.40, fair consistency: kappa value 0.41–0.60, substantial consistency: kappa value 0.61–0.80, complete consistency: kappa value 0.81-1.00 ). Survival analysis was conducted using the Kaplan-Meier method, and survival curves were plotted. The comparison of survival rates between the two groups was performed using the log-rank test. The significance level was set at α = 0.05.

## Results

### Clinicopathological features

NGS was conducted in 357 liquid-based cytology samples collected from patients with lung adenocarcinoma including 157 males (44.0%) and 200 females (56.0%), with a median age of 61 years. The majority of cases were classified as stage IV according to the AJCC clinical staging system (264/357, 73.9%). Stage III accounted for 33 cases (9.2%), while the remaining 60 cases could not be clinically evaluated. The main sources of specimens were pleural effusion samples (185/357, 51.8%) and fine-needle aspiration cytology samples (141/357, 39.5%). The remaining samples included a small number of fiberoptic bronchoscopy biopsies (29/357, 8.1%) and sputum specimens (2/357, 0.6%). The samples were divided into two groups based on whether or not they had received EGFR-TKIs targeted therapy before NGS test: cohort 1 included 275 TKI-naive cases and cohort 2 included 82 TKI-treated cases.

### Molecular profiling

**Fig. 1:**
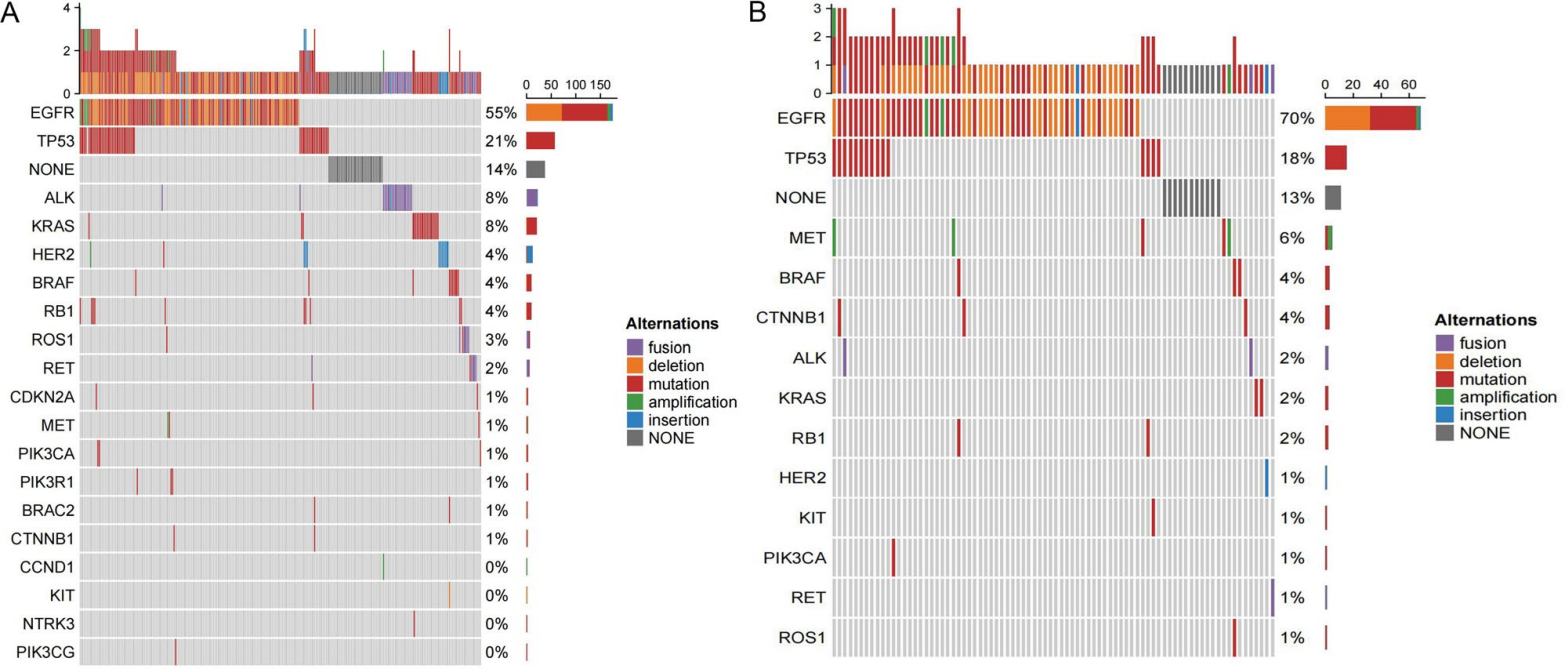
The molecular profiling of cohort 1 (**A**) and cohort 2 (**B**). *NONE means specimen has no gene mutation detected

237 samples from the cohort 1 (86.2%) were found to have cancer-related gene alterations. 71 specimens from the cohort 2(86.6%) were found to have cancer-related gene alterations. *EGFR* was the most frequently mutated gene in both cohorts, with a greater mutation rate in cohort 2 compared to cohort 1 (69.5% > 54.9%), which was statistically significant (*P* = 0.019). The T790M resistance mutation rate was higher in cohort 2 than in cohort 1 (13.4% > 8.0%), however this difference was not statistically significant (*P* = 0.137). Additionally, we discovered that cohort 2 had a statistically significant increase in the frequency of *MET* mutation (6.1% > 1.1% *P* = 0.024). In cohort 2 the most common mutation status was *MET* amplification (3/5, 60%), while it differs in the cohort 1 (Table 1). The molecular profiling of each cohort can be seen in Table 2 and Fig. 1, specific mutation details can be seen in Supplementary Table 1.

**Table 1:** Comparison of *EGFR* and *MET* mutation status in cohort 1 and cohort 2

Number of specimen	TKI-treatment＊	EGFR	Mutation	MET	Mutation
7	1	0		14	3028 + 3 A > T
17	1	21	L858R	amplification	
58	1	19	DEL	amplification	
81	1	19	DEL	amplification	
82	1	19	DEL	15	V1070A
83	0	0		14	D963fs
84	0	0		amplification	
175	0	0		14	D1010N

＊For TKI-treatment, ‘1’ represents for ‘TKI-treated’, ‘0’ stands for ' TKI-naive’

**Table 2:** The NGS results of cohort 1 and cohort 2

	TKI-naive (275 cases)	TKI-treated (82 cases)	*p* value
EGFR	54.9%(151/275)	69.5%(57/82)	0.019*
Sensitizing EGFR	50.9%(140/275)	63.4%(52/82)	0.046*
EGFR T790M	8.0%(22/275)	13.4%(11/82)	0.137
KRAS	7.6%(21/275)	2.4%(2/82)	0.092
BRAF	3.6%(10/275)	3.7%(3/82)	1.000
ALK	7.6%(21/275)	2.4%(2/82)	0.092
ROS-1	2.5%(7/275)	1.2%(1/82)	0.774
RET	2.2%(6/275)	1.2%(1/82)	0.922
MET	1.1%(3/275)	6.1%(5/82)	0.024*
HER2	4.4%(12/275)	1.2%(1/82)	0.318

### Consistency comparison

We collected 22 paired histological specimens with LBC specimens. The gene testing results of LBC specimens were completely consistent with the histological specimens in 72.7% (16/22) of patients. The concordance rate for *EGFR* was 95.5% (21/22, kappa = 0.861, *p* < 0.001). The concordance rates for *KRAS, BRAF*, and *ALK* were all 100% and no mutations were detected. The concordance rates for *ROS-1* and *RET* were both 100%, with mutation rates of 4.5% (1/22) for both. The concordance rate for *MET* was 81.8% (18/22). The concordance rate for *HER2* was 90.9% (20/22). Among the 6 inconsistent specimens, the main genes involved were *MET* and *HER2*. In 3 cases, only the histological specimens showed *MET* mutation, while in 1 case, only the cytological specimen showed *MET* mutation. In 1 case, only the histological specimen showed *HER2* mutation, and in 1 case, only the cytology specimen showed *HER2* mutation.

### Tumor cellularity

All LBC specimens were diagnosed as lung adenocarcinoma by two cytopathologists, and the tumor cellularity was evaluated. Based on the assessment results, all samples were divided into three groups: Group 1 (G1): 10%~19%; Group 2 (G2): 20%~30%; Group 3 (G3): >30%.

**Fig. 2:**
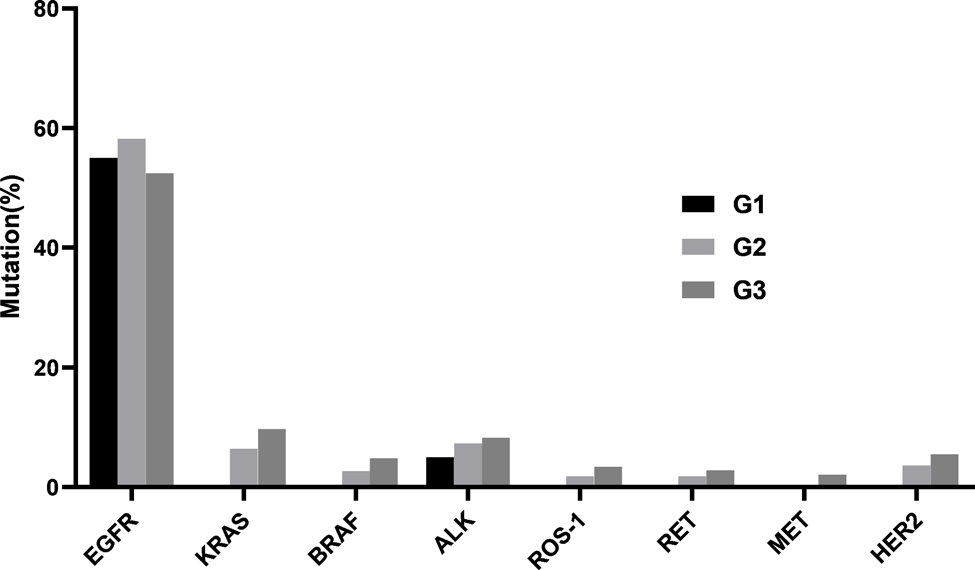
Comparison of mutation frequencies in different tumor cellularity groups in cohort 1 (G1: samples with 10–19% tumor cellularity; G2: 20–30% tumor cellularity; and G3: >30%tumor cellularity)

In cohort 1, there were 20 cases in G1, 110 cases in G2, and 145 cases in G3. The frequencies of *EGFR* mutation in the three groups were similar (55.0% vs. 58.2% vs. 52.4%). For the other mutated genes, the mutation frequencies showed a pattern of G3 > G2 > G1, but there were no statistically significant differences between any two groups (Fig. 2).

**Fig. 3:**
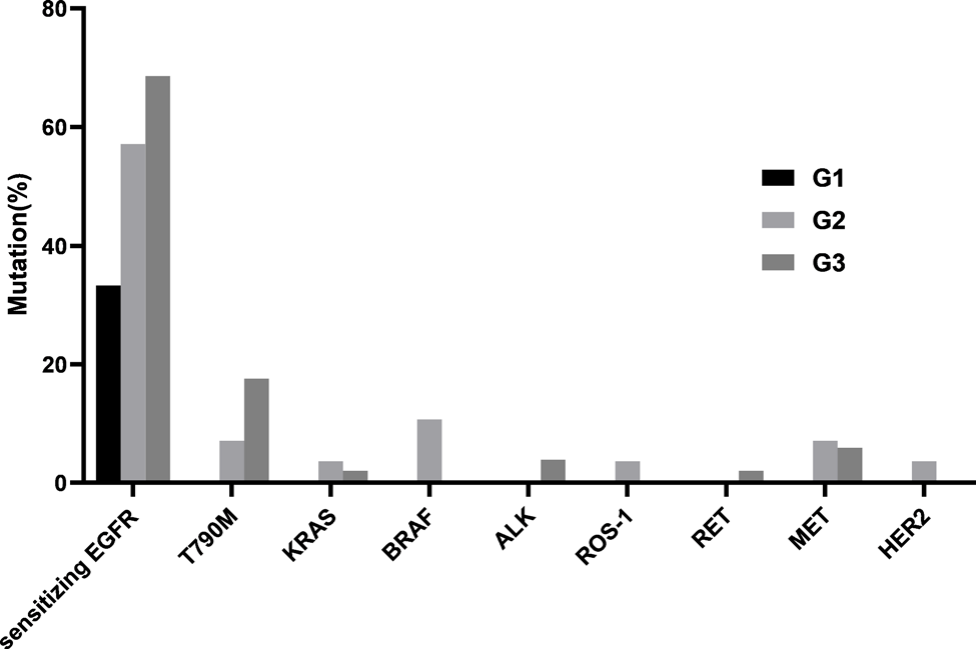
Comparison of mutation frequencies in different tumor cellularity groups in cohort 2 (G1: samples with 10–19% tumor cellularity; G2: 20–30% tumor cellularity; and G3: >30%tumor cellularity)

In cohort 2, there were 3 cases in G1, 28 cases in G2, and 51 cases in G3. The frequency of *EGFR* sensitizing mutation showed a pattern of G3 > G2 > G1, but there were no statistically significant differences between any two groups (G2 vs. G1, 57.1% vs. 33.3%, *P*=0.576; G3 vs. G2, 68.6% vs. 57.1%, *P*=0.307; G3 vs. G1, 68.6% vs. 33.3%, *P*=0.255). The frequency of T790M mutation also showed a pattern of G3 > G2 > G1, however there were no statistically significant differences between any two groups (G2 vs. G1, 7.1% vs. 0%, *P*=1.000; G3 vs. G2, 17.6% vs. 7.1%, *P*=0.342; G3 vs. G1, 17.6% vs. 0%, *P*=1.000). For the other mutated genes, there were no statistically significant differences between any two groups (Fig. 3).

### 3.5. Evaluation of TKI treatment

**Fig. 4:**
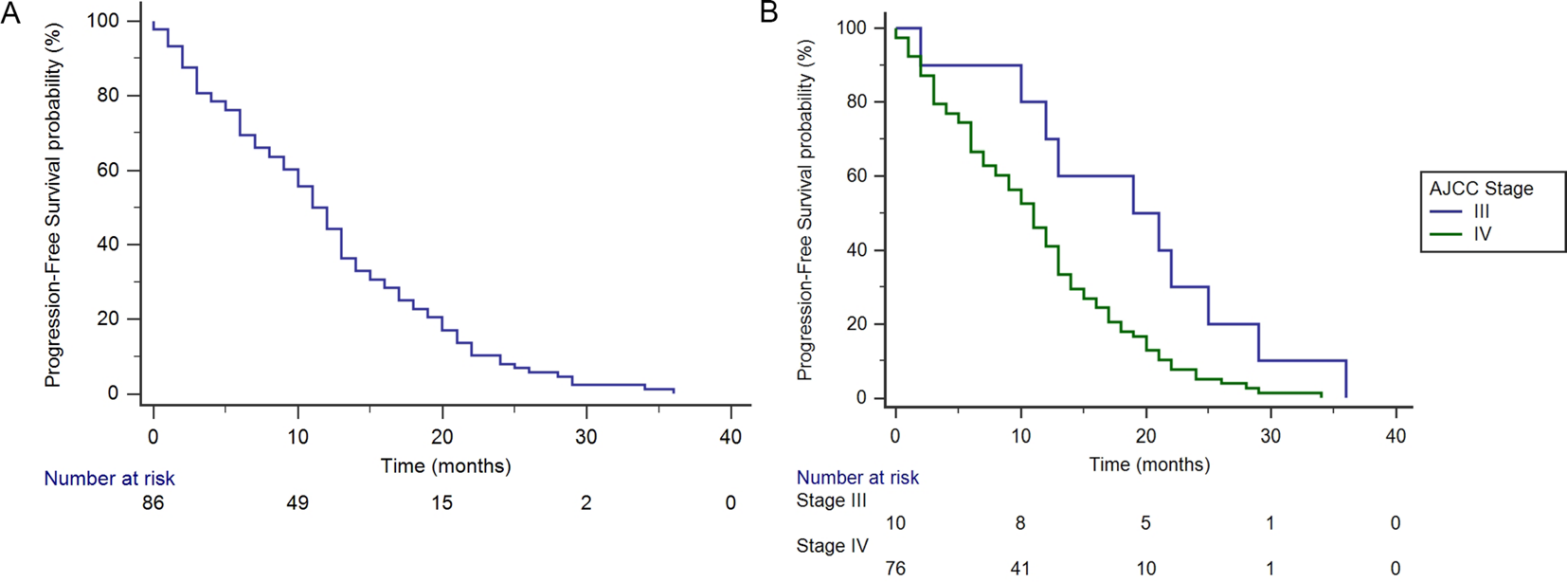
(**A**) PFS of *EGFR*-mutant patients. (**B**) PFS of *EGFR*-mutant patients between stage III and stage IV

The efficacy of EGFR-TKIs was assessed in 92 patients with *EGFR* mutation who had complete clinical follow-up data. 88 of the 92 patients had disease progression, with a median PFS of 12.1 months (95% CI: 10.4–13.9) (Fig. 4A). The median PFS and clinical stage were significantly correlated in the group of 92 patients with *EGFR* mutation (χ^2^ = 6.1773, *p* = 0.0129). Among the 88 patients who experienced disease progression, 10 were in clinical stage III, with a median PFS of 18.7 months (95% CI: 13.1–24.3), while 78 were in stage IV, with a median PFS of 11.3 months (95% CI: 9.6–13.0) (Fig. 4B). The median PFS of patients in the *EGFR* mutation group was not associated with whether they received targeted therapy (χ^2^ = 0.08161, *p* = 0.7751) or the presence of *EGFR* exon 20 mutation (χ^2^ = 2.4196, *p* = 0.1198).

## Discussion

Molecular profiling is a requirement for implementing precision therapy for NSCLC, which has grown quickly in recent years [5]. There is a global trend toward the use of techniques that are less invasive and discomfort-free for patients when collecting specimens for genetic testing. Cytological specimens offer the advantages of being simple to obtain, inflicting less trauma to patients, and having high reproducibility compared to histological specimens. Additionally, liquid-based cytological specimens can be kept for a very long time in storage. Cytological specimen is a good choice for genetic testing, particularly for patients with advanced NSCLC.

Although NGS has been rapidly adopted in molecular diagnostics, there are still some obstacles that must be carefully evaluated in the quality control process of each step, such as variations in tumor cellularity, heterogeneity between primary and metastatic tumors, and multifocality [7]. Wen et al. [10] discovered that 73.9% of NSCLC patients in China had at least one targetable gene mutation suggested by the National Comprehensive Cancer Network guidelines, which includes *EGFR, ALK, ERBB2, MET, BRAF, RET* and *ROS1, EGFR* mutation accounts for 50.1%. Amplification-based NGS was used in this study to detect a total of 357 samples of liquid-based cytology from lung adenocarcinoma, including 82 TKI- treated specimens and 275 TKI-naive specimens. The cancer-related gene alterations reported in the two cohorts were similar (86.2% vs. 86.6%), with *EGFR* having the highest mutation frequency in both. The TKI-treated cohort’s *EGFR* mutation rate was higher than that of the TKI-naive specimens (69.5% > 54.9%, *p* = 0.019). Previous studies have shown that most patients who initially respond to EGFR-TKIs progress by acquiring the EGFR T790M mutation, which is the most common resistance mechanism [11]. This study also found that the frequency of the T790M resistance mutation was significantly higher in the TKI-treated cohort than in the initial diagnosis samples (13.4% > 8.0%). In NSCLC, the three main mechanisms of *MET* dysregulation include protein overexpression, exon 14–skipping mutations, or gene amplification [12]. *MET* amplification have emerged as the resistance mechanism to EGFR-TKIs in addition to T790M mutation [13]. Initial data in *EGFR*-mutant tumors reported that *MET* amplification was detected in 5–22% of NSCLC with acquired resistance to first-generation EGFR- TKIs [14]. Our study also found the frequency of the *MET* mutation was significantly higher in the TKI-treated cohort than in the initial diagnosis samples (6.1% > 1.1% *P* = 0.024), and *MET* amplification accounted for 3.7% in the TKI-treated group (3/82, 3.7%). Several preclinical trials in *EGFR*-mutant tumors and acquired *MET* amplification reported the efficacy of dual EGFR-TKIs plus MET inhibitor therapy [15]. Two of those three cases with *MET* amplification in our study combined EGFR-TKIs with MET inhibitor (crizotinib) in the latter treatment and were still in the follow-up. Several anti–MET-targeted therapies (exon 14 skipping mutation) have been approved recently and *MET* exon 14 alterations are generally thought to be mutually exclusive with other major driver mutations [15, 16]. In this study, we also found three cases with exon 14 alterations, none of them gained other driver mutations. For case 7, patient was found adenocarcinoma in the upper lobe of left lung and treated, then found another tumor in the upper lobe of right lung with *MET* exon 14 alteration and treated with crizotinib. The rest two case with *MET* exon 14 alteration never received TKI treatment before, one of them treated with crizotinib, the other hadn’t treatment record.

There were 22 histologically matched specimens in the present study. The histology specimens and the gene testing results of the liquid-based cytology samples from 72.7% of patients were entirely consistent. In this group, the *EGFR* consistency rate was 95.5% (21/22, Kappa = 0.861, *p*<0.001). The consistency rates of *KRAS, BRAF, ALK, ROS-1*, and *RET* were all 100%, proving the reliability of gene testing results in liquid-based cytology. The inconsistent specimens mainly manifested in the *MET* and *HER2* genes, mainly due to low tumor cellularity, which made accurate interpretation impossible. Evaluating tumor cellularity is a necessary process for molecular testing. Generally, the minimum recommended value for tumor cellularity in routine mutation testing is more than twice the limit of detection (LOD) [17]. In this study, the LOD of the NGS platform was 5%. Therefore, we performed NGS testing on liquid-based cytology specimens of lung adenocarcinoma with ≥ 10% tumor cellularity. In cohort 1, there was no significant difference in the frequency of *EGFR* mutation among different tumor cellularity. However, in cohort 2, the frequency of *EGFR* sensitizing mutation and T790M resistance mutation in G1 (10%~19% tumor cellularity ) was significantly lower than in G2 (20%~30% tumor cellularity) and G3 (>30% tumor cellularity). The cutoff threshold of tumor cellularity for NGS testing in earlier research was 20% [18]. Recently Gu et al. [19] found that only in surgical samples, the frequency of *EGFR* mutation in samples with tumor cellularity < 10% was significantly lower than in samples with tumor cellularity ≥ 10%. In biopsy specimens and cytology specimens, the frequency of *EGFR* mutation in samples with tumor cellularity < 20% was comparable to specimens with tumor cellularity ≥ 20%, and the low tumor cellularity was not associated with mutation rates of *ALK, ROS1, BRAF, KRAS, RET, HER2, CMET, NRAS*, and *PIK3CA*. However, Li et al. found that the frequency of T790M mutation in specimens with tumor cellularity < 20% after TKI drug treatment was significantly lower than that in specimens with tumor cellularity ≥ 20% [7]. Therefore, our study suggests that for initial diagnosed patients, NGS testing can still be attempted in liquid-based cytology specimens with tumor cellularity less than 20% in order to explore the possibility of targeted therapy. However, for patients who require adjustment of treatment after targeted therapy, a tumor cellularity of ≥ 20% is needed to determine the presence of acquired resistance mutations, such as T790M and *MET* amplification.

Multiple large-sample clinical studies have shown that in advanced NSCLC patients with *EGFR* mutation detected by histological specimens, the median PFS can reach 9.2 to 18.9 months when treated with EGFR-TKIs [20–22]. However, there is a lack of large-sample clinical studies evaluating the efficacy of EGFR-TKI treatment in *EGFR* mutation-positive patients using liquid-based cytology specimens with NGS testing. In a previous study, we evaluated the efficacy of RT-PCR testing on liquid-based cytology specimens in lung adenocarcinoma patients and found that the disease control rate (DCR) and median PFS (89.0% and 13.8 months, respectively) in patients with *EGFR* gene mutation were higher than those in patients without *EGFR* gene mutation (30.8% and 1.4 months, respectively) [23]. Lu et al. [24] divided 132 advanced NSCLC patients into a group with EGFR mutation (72 cases) and another group without *EGFR* mutation (60 cases) in pleural fluid cell blocks. The median progression-free survival (PFS) after EGFR-TKI treatment was 11 months in the positive group and 1 month in the negative group, with a statistically significant difference (*P* < 0.05). In this study, we observed 92 *EGFR*-mutant lung adenocarcinoma patients treated with EGFR-TKI, of which 88 cases experienced progression. The median PFS was 12.1 months, consistent with previous literature reports, once again demonstrating the consistency between liquid-based cytology NGS testing results and the efficacy of tissue-based specimens in guiding targeted therapy for lung adenocarcinoma patients.

There are still several limitations to this study: (1) There are few samples available following TKI treatment, and they are not well matched to the non-targeted treatment group. Further research is needed to expand the sample size. (2) The study only included a small number of samples with tumor cellularity less than 20%, making comparisons between the various groups insufficient. (3) There is a lack of reporting time limitations comparability. For liquid-based cytology and histology samples examined by NGS in the future, it will be possible to compare the time from sample collection to report issue in order to give more prompt and thorough testing techniques for clinical selection.

In summary, this study recommends that, prior to NGS testing, the tumor cellularity in liquid-based cytology specimens should be carefully assessed. Specimens having a tumor cellularity of less than 20% may be tested on initial diagnosed patients. However, for patients with recurrent disease after treatment, it is recommended to use samples with tumor cellularity ≥ 20%. Additionally, this study supports liquid-based cytology specimens are of vital importance in NGS testing for patients with lung adenocarcinoma since they make it easy to identify acquired mutations and provide useful clinical diagnostic and therapy advice.

## Conclusions

Cytological specimens are important sources for gene detection of advanced lung adenocarcinoma. When using LBC specimens for molecular testing, it is recommended to fully evaluate the tumor cellularity of the specimens.

### Electronic supplementary material

Below is the link to the electronic supplementary material.

Supplementary Material 1

## Data Availability

The datasets during the current study are not publicly available due to it containing information that could compromise the privacy of research participants but are available from the corresponding author on reasonable request.

## Abbreviations

LBC: Liquid-based cytology
NGS: Next-generation sequencing
NSCLC: Non-small cell lung cancer
EGFR: Epidermal growth factor receptor
TKI: Tyrosine kinase inhibitor
OS: Overall survival
PFS: Progression-free survival
AJCC: American Joint Committee on Cancer
FNA: Fine needle aspiration
LOD: The limit of detection
DCR: Disease control rate
